# ZYNQ-Based Visible Light Defogging System Design Realization

**DOI:** 10.3390/s24072276

**Published:** 2024-04-03

**Authors:** Bohan Liu, Qihai Wei, Kun Ding

**Affiliations:** 1 Shanghai Institute of Technical Physics, Chinese Academy of Sciences, Shanghai 200083, China; 2 University of Chinese Academy of Sciences, Beijing 100049, China

**Keywords:** defogging, dark channel prior, image segmentation, ZYNQ, visible light

## Abstract

Under a foggy environment, the air contains a large number of suspended particles, which lead to the loss of image information and decline of contrast collected by the vision system. This makes subsequent processing and analysis difficult. At the same time, the current stage of the defogging system has problems such as high hardware cost and poor real-time processing. In this article, an image defogging system is designed based on the ZYNQ platform. First of all, on the basis of the traditional dark-channel defogging algorithm, an algorithm for segmenting the sky is proposed, and in this way, the image distortion caused by the sky region is avoided, and the atmospheric light value and transmittance are estimated more accurately. Then color balancing is performed after image defogging to improve the quality of the final output image. The parallel computing advantage and logic resources of the PL (Programmable Logic) part (FPGA) of ZYNQ are fully utilized through instruction constraints and logic optimization. Finally, the visible light detector is used as the input to build a real-time video processing experiment platform. The experimental results show that the system has a good defogging effect and meet the real-time requirements.

## 1. Introduction

With the rapid development of image processing and image analysis technology, image defogging technology, as one of the important research directions, is more and more widely used in real-time monitoring, target identification and target detection [1]. In practical applications, under a foggy environment, the air contains a large number of suspended particles, the reflected light on the surface of the object is attenuated, and the atmospheric light is mixed into the imaging light, resulting in the loss of acquired image information, contrast degradation, and the subsequent processing and analysis of difficulties.

At this stage, most of the research on defogging algorithms for single images is based on the Central Processing Unit (CPU) or Graphics Processing Unit (GPU). There are shortcomings, such as high hardware cost, large size, high power consumption, and poor real-time performance [2].

The emergence of FPGA (Field Programmable Logic Gate Array) has brought new ideas for digital image processing. A complete digital image processing system not only has high requirements for computing power, but also needs complete system peripherals to fulfill complex control functions. The ARM processor is a mature embedded system processor, and the FPGA has high-speed parallel computing capability. Therefore, the ARM + FPGA solution is very suitable for the design of digital image processing systems with hardware-level computing speed and low power consumption.

In order to improve the defogging ability and real-time performance of the video defogging algorithm, this article starts from the dark channel defogging algorithm, improves the existing algorithm, and proposes a specific scheme for the algorithm to be deployed on the programmable logic architecture.

## 2. Defogging Algorithm Overview

### 2.1. Foggy Day Imaging Model and Dark Channel Defogging Algorithm

Narasimhan and Nayar [3,4] found that as light passes through the air into the imaging device, suspended atmospheric particles (e.g., water vapor) adsorb and scatter the reflected light from the target, resulting in attenuation of the reflected light from the target. The ambient light around the target also affects the imaging. As a result, the atmospheric scattering model is proposed.

In this model, physical modeling of fog images is defined as follows:

(1) $$E\left(d,\lambda \right)=E_{0}\left(\lambda \right)e^{-\beta \left(\lambda \right)d}+L_{h}\left(\infty ,\lambda \right)\left(1-e^{-\beta \left(\lambda \right)d}\right)\;$$

E(d,λ) denotes the spectral irradiance at wavelength λ for an object at distance d from the detection device. β(λ) is the total scattering coefficient.$\;E_{0}\left(\lambda \right)$ is the irradiance at the source. $E_{0}\left(\lambda \right)e^{-\beta \left(\lambda \right)d}$ is the intensity of the incident light reaching the detection device after attenuation. $L_{h}\left(\infty ,\lambda \right)$ is atmospheric light radiance, which is observable. $L_{h}\left(\infty ,\lambda \right)\left(1-e^{-\beta \left(\lambda \right)d}\right)$ is the light intensity of the attenuated ambient light reaching the detection device.

A simplified model of this model is widely used in computer vision and computer graphics for fog image formation studies:

(2) I(x)=J(x)t(x)+A(1−t(x)) 

I(x) is the existing image (the image to be defogged) and J(x) is the fog-free image to be recovered. A is the global atmospheric light component, i.e., the intensity of light at infinity. t(x) is the transmittance, reflecting the light’s ability to penetrate through the fog, which is determined by the distance between the scene and the camera.

For this model, He [5] proposed a dark channel defogging algorithm based on a priori knowledge. That is, in the vast majority of non-sky localized regions, some pixels will always have at least one color channel with a very low value. That is, the minimum value of light intensity in that region is extremely small. For any image, the mathematical definition of its dark channel is expressed as follows:

(3) $$J^{dark}\left(x\right)=\underset{y\in \Omega \left(x\right)}{\min}\left(\underset{c\in \left\{r,g,b\right\}}{\min}J^{c}\left(y\right)\right)$$

$J^{c}$ denotes some color channel of the color image and Ω(x) denotes the window region centered on pixel *x*. $J^{dark}$ is the dark channel image. Dark channel a priori theory states

(4) $$J^{dark}\left(x\right)\to 0$$

Analyzing the model of Equation (2) using dark channel theory yields a prediction of the transmittance t(x) as

(5) $$t\left(x\right)=1-\omega \underset{y\in \Omega \left(x\right)}{\min}(\underset{c}{\min}\frac{I^{c}\left(y\right)}{A^{c}})$$

ω is the fog weight factor which is used to preserve the effect of fog on distant objects and the depth of field to make the image more realistic. The algorithm selects the brightest 0.1% pixel in the dark channel image as the global atmospheric light value A.

In summary, the image defogging model is formulated as follows:

(6) $$J\left(x\right)=\frac{I\left(x\right)-A}{\max\left(t\left(x\right),t_{0}\right)}+A$$

where $t_{0}$ is a limiting factor to avoid a large J value due to too small a transmittance and to prevent the overall image from overshooting to the white field. It is usually taken as 0.1.

### 2.2. Limitations of Dark Channel Defogging

**Figure 1:**
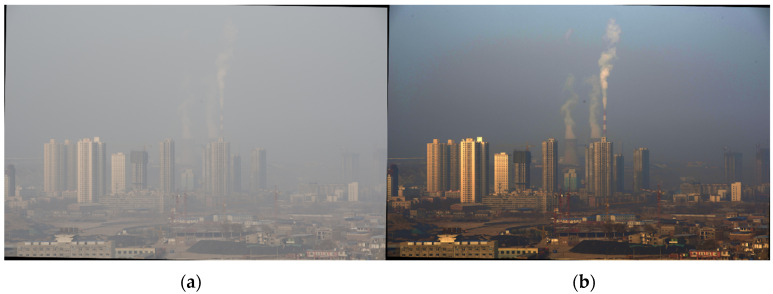
Dark channel defogging effect: (**a**) images with sky areas; (**b**) result of dark channel algorithm.

In the dark channel algorithm-processed image, the brightness of the screen is significantly reduced, and it is easy for the sky area to contain the halo phenomenon and color distortion [6], as shown in Figure 1. Foggy images often contain sky area. Therefore, it should be improved to counteract this problem.

### 2.3. Causes of Color Distortion in the Dark Channel Algorithm

After a foggy sky image is processed by the basic dark channel algorithm, some areas show reduced brightness and image color distortion. This phenomenon is particularly noticeable in the sky region. The reason for this problem is that the estimation algorithm of atmospheric light A and the transmittance t(x) is too simple. High pixel values at certain locations in the image, such as bright reflections and artificial light sources, can lead to incorrect estimates of atmospheric light A. The transmittance is calculated from A and is equally inaccurate. Even when the atmospheric light is estimated correctly, the pixel values in the sky regions are high and do not converge to zero. The priori theory of the dark channel does not apply in these regions, i.e., Equation (4) does not hold. That is to say,

(7) $$J^{dark}\left(x\right)=\underset{y\in \Omega \left(x\right)}{\min}\left(\underset{c\in \left\{r,g,b\right\}}{\min}J^{c}\left(y\right)\right)\neq 0$$

The exact transmittance function is derived directly from Equation (2).

(8) $$t\left(x\right)=\frac{1-\omega \underset{y\in \Omega \left(x\right)}{\min}(\underset{c}{\min}\frac{I^{c}\left(y\right)}{A^{c}})}{1-\omega \underset{y\in \Omega \left(x\right)}{\min}(\underset{c}{\min}\frac{J^{c}\left(y\right)}{A^{c}})}$$

**Figure 2:**
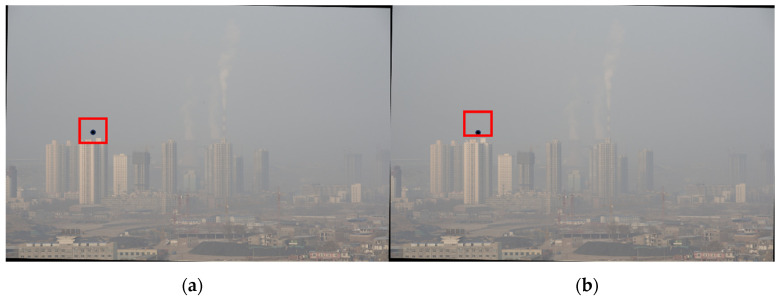
Different windows for transmittance calculation: (**a**) window before adjustment; (**b**) window after adjustment.

According to Equation (7), $\underset{y\in \Omega \left(x\right)}{\min}(\underset{c}{\min}\frac{J^{c}\left(y\right)}{A^{c}})$ cannot be approximated to be 0, and the denominator of Equation (8) is less than 1. The actual transmittance is larger than the transmittance estimated according to the a priori theory of the dark channel. At the same time, rough estimation of transmittance can also make the dehazing image appear with the halo phenomenon. This is especially true at strong boundaries, such as the sky–scene junction. This is because t(x) is actually determined by the scene distance, and the distance of the scene at the junction varies so much that it is inaccurate to estimate the same t(x) in a window containing two depths of field [7]. For example, the color of the building on the left in Figure 1b appears abnormal. This is due to the fact that the transmittance calculation window contains both the sky and the scene at different depth-of-field positions. As shown in Figure 2, the calculation window needs to be adjusted. Both the atmospheric light and transmittance estimation methods in the dark channel algorithm need to be improved, and in particular the sky region needs to be segmented.

The black point in Figure 2 is the target pixel. We intend to calculate the transmittance at that pixel. The red window is the window for calculating the transmittance. We utilize all pixels within the window to calculate the transmittance. The window in subfigure (a) includes both the sky and the building, and this choice of window is inaccurate. The window in subfigure (b) is adjusted to include only the sky region. This adjustment results in a more accurate transmittance estimate.

Many researchers have improved and supplemented the dark channel algorithm, such as improving the dark channel algorithm by adding filtering algorithms as a complement to the dark channel algorithm, like the soft matting method [8], guided filtering [9], joint bilateral filtering [10], etc., which also achieve the optimized defogging effect. However, these methods are computationally huge, and it takes too long to process a single image. Real-time processing cannot be realized. In order to improve the speed of the algorithm, some researchers have tried to deploy some defogging algorithms on FPGA platforms, such as SSR and MSR algorithm defogging based on the “Retinex” theory [11,12]. These algorithms can meet the real-time requirements, but the defogging effect still needs to be improved.

## 3. Algorithmic Principle

**Figure 3:**
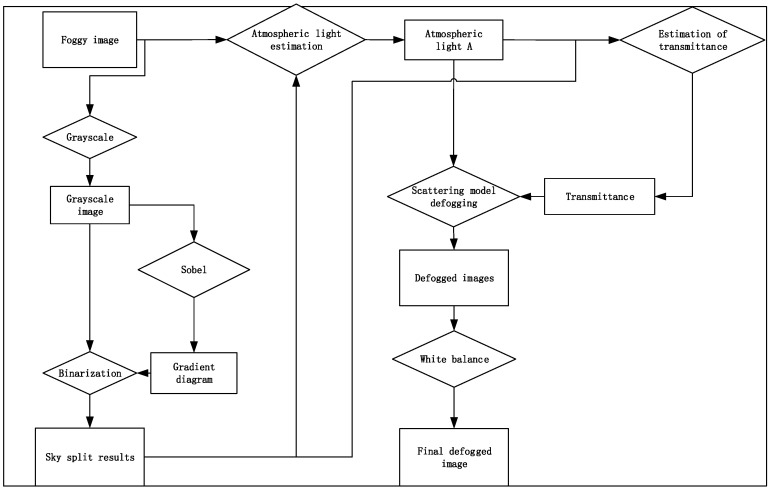
Defogging algorithm flow.

In this article, an optimized defogging algorithm based on the dark channel defogging model is proposed. The gradient and grayscale characteristics of the sky region are utilized for sky region segmentation, based on which the atmospheric light A and transmittance t(x) in the atmospheric scattering model are corrected. The image is defogged using the correction parameters. Finally, color correction is performed using the white balance algorithm. While improving the defogging effect, processed images are easier for the human eye to view. Finally, the algorithm is improved based on the circuit structure of ZYNQ and deployed on the ZYNQ platform. After experimental testing, the algorithm has good defogging results. The algorithm architecture is shown in Figure 3.

### 3.1. Sky Segmentation

There are some defogging algorithms that attempt to split the sky region. For example, Zhang [13] calculates the probability that a pixel point belongs to the sky region based on the position of the pixel point in the image. A separate calculation for each pixel leads to a slow speed. Therefore, looking for the characteristics of the sky region as a whole is a quicker way to segment it.

The sky region in the image is more uniform in color, has a smaller gradient, and has a larger gray value. Sky segmentation can be performed based on this feature. The segmentation method is as follows:

- Convert an RGB image into a grayscale map.
- Compute the image gradient using the Sobel operator.
- Segment the image based on the image gradient and gray value binarization, where the gradient value is less than the threshold $\theta_{1}$ and the gray value is greater than $\theta_{2}$.
- Mark the image pixels that satisfy the threshold as sky regions and perform morphological filtering on the image to segment the sky regions.

**Figure 4:**
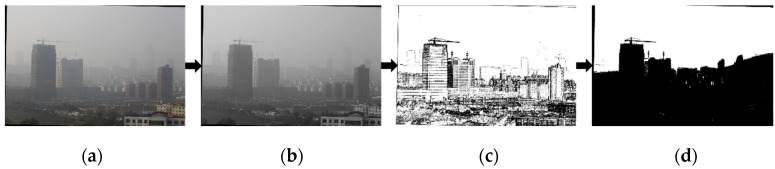
Sky area split process: (**a**) input image; (**b**) grayscale image; (**c**) gradient image; (**d**) segmental result.

The segmentation process is shown in Figure 4.

### 3.2. Parameter Corrections

After segmenting the sky region of the image, atmospheric light A and transmittance t(x) are computed.

#### 3.2.1. Atmospheric Light Estimation

After determining the sky area, atmospheric light estimation is performed. In order to avoid the effect of overly bright pixel points, the average, maximum, and minimum values of the pixels within the small window are calculated simultaneously based on the characteristic of insignificant changes in brightness values in the sky region. Calculate:

(9) $$K_{\Omega \left(x\right)}=\underset{y\in \Omega \left(x\right)}{\mathrm{average}}(\underset{c}{I^{c}\left(y\right)})-(\underset{y\in \Omega \left(x\right)}{\max}(\underset{c}{I^{c}\left(y\right)})-\underset{y\in \Omega \left(x\right)}{\min}(\underset{c}{I^{c}\left(y\right)}))$$

The result of Equation (9) is used as an evaluation parameter to find the region with the highest value of the evaluation parameter in the sky region, where the color vector is selected such that $\left|\left|\left(I_{r}\left(x\right),I_{g}\left(x\right),I_{b}\left(x\right)\right)-\left(255,255,255\right)\right|\right|$ is minimized as the atmospheric light A. This avoids the problem of overly bright light spots in the sky region.

#### 3.2.2. Estimation of Transmittance

The dark-channel a priori assumption for non-sky regions holds, and the transmittance estimation method remains unchanged. According to the previous analysis, the actual transmittance of the sky region transmittance is larger than the transmittance estimated by the dark channel a priori theory. The sky area transmittance is corrected to

(10) $$t\left(x\right)=\max\left[\frac{Q}{\left|I\left(x\right)-A\right|},1\right]\cdot \left[1-\omega \underset{y\in \Omega \left(x\right)}{\min}(\underset{c}{\min}\frac{I^{c}\left(y\right)}{A^{c}})\right]$$

Introducing Q defined as a tolerance, the value of |I(x)−A| is larger in the sky region than in the non-sky region. In the sky area, $\frac{Q}{\left|I\left(x\right)-A\right|}>1$, which is used to adjust the sky region transmittance.

**Figure 5:**
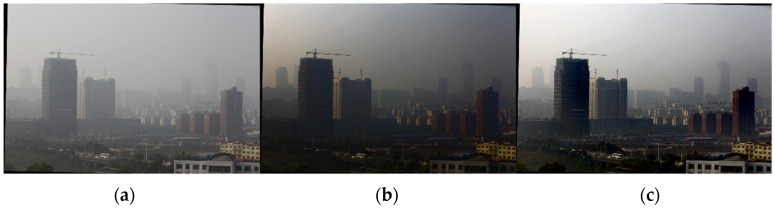
Defogging effect before and after parameter correction: (**a**) original figure; (**b**) result of dark channel defogging; (**c**) defogging results after parameter correction.

Using the above calculated atmospheric light and transmittance according to the dark channel defogging model for defogging processing, a preliminary defogged image is obtained, as shown in Figure 5.

#### 3.2.3. Color Correction

**Figure 6:**
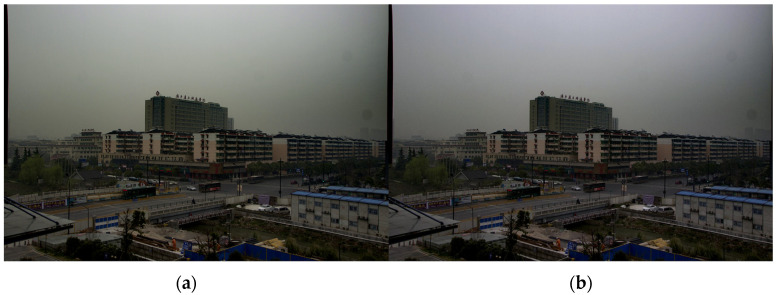
Comparison of images before and after white balance: (**a**) initial defogging image; (**b**) white balance image.

The automatic white balance (AWB) algorithm can eliminate the appearance of bias color problems in different light conditions, simulating the color constancy of the human visual system. Commonly used AWB algorithms are the grayscale world method, perfect reflection method, etc. [14]. In this article, we choose to consume less resources of the grayscale world method for the image white balance, to ensure that the algorithm is effective in recovering the original color and color temperature of the image. The algorithm is implemented as follows:

(11) $${\left\lceil \begin{array}{c} R\\ G\\ B \end{array}\right\rceil }_{AWB}=\left[\;\begin{array}{ccc} r_{g} & 0 & 0\\ 0 & g_{g} & 0\\ 0 & 0 & b_{g} \end{array}\right]{\left\lceil \begin{array}{c} R\\ G\\ B \end{array}\right\rceil }_{in}$$

(12) $$\{\begin{array}{c} r_{g}=K/R_{avg}\\ g_{g}=K/G_{avg}\;\\ b_{g}=K/B_{avg} \end{array}$$

where $R_{avg}$, $G_{avg}$, $B_{avg}$ are the three channel luminance averages of the RGB image, respectively. $K=\left(R_{avg}+G_{avg}+B_{avg}\right)/3$, or is taken as half of the maximum value of each channel: 128. In this paper, the first calculation method is chosen. The AWB algorithm can recover some of the color information lost due to the defogging algorithm. Figure 6 shows the comparison before and after AWB algorithm processing.

## 4. ZYNQ-Based Hardware Implementation

### 4.1. System Design Architecture

**Figure 7:**
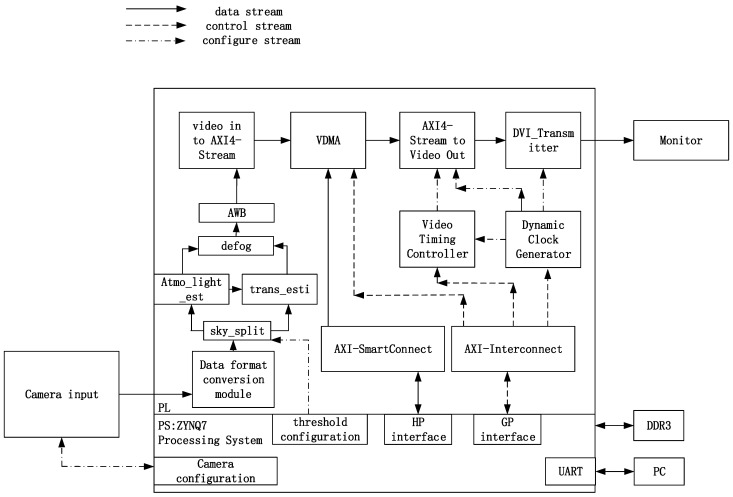
Structure of image defogging processing system.

This article adopts the ZYQN platform, which integrates an ARM Processing System (PS) and FPGA Programmable Logic (PL). The PS part completes the image acquisition and image storage functions, and the PL part completes the defogging algorithm and display output functions. PL and PS interact with each other through the high-speed bus AXI4, which greatly improves the execution efficiency and meets the real-time processing requirements [15]. PL and PS interact with each other through the high-speed bus AXI4, which greatly improves the execution efficiency and meets the real-time processing requirements. The overall hardware architecture is shown in Figure 7.

The camera inputs video data to the PL section. The data format conversion module converts the video stream into the HLS::Mat format for easy processing by ZYNQ. After the video image has completed sky segmentation, atmospheric light and transmittance are calculated and then defogged. After AWB, the signal is converted into the AXI4 data stream for output to the monitor. The host computer can use serial commands to adjust the algorithm parameters for better results.

### 4.2. FPGA Design of Image Defogging Module

The defogging module is divided into different sub-functional modules according to their functions. As shown in Table 1.

#### 4.2.1. Design of Sky Area Segmentation Module and Calculation of Atmospheric Light Values

**Figure 8:**
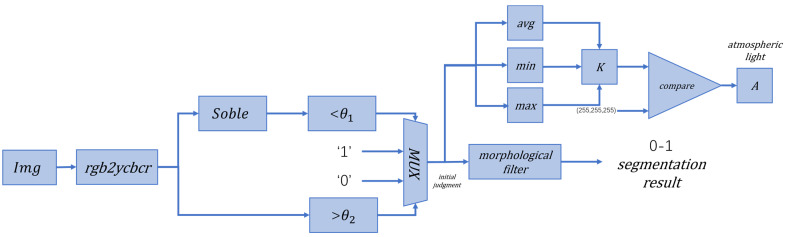
Sky segmentation and atmospheric light estimation architecture.

Firstly, the input image is grayed out, the image gradient is obtained by the Sobel operator, and binarization is performed by combining the image gray value with a preset threshold. The pixels marked with “1” are initially recognized as the sky region. The fused sky is then obtained using morphological filtering. At the same time, the sky region is evaluated using the mean, maximum, and minimum operators to obtain the evaluation parameter K in Equation (9), and the color vector of the largest region of K is obtained as the atmospheric light A. Figure 8 shows the module architecture.

#### 4.2.2. Transmittance Module Design

**Figure 9:**
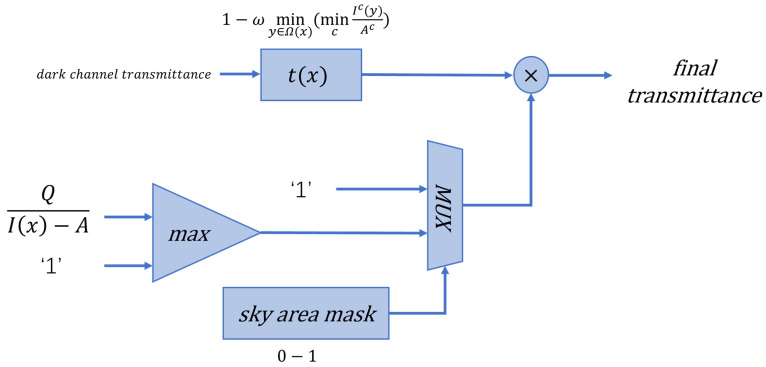
Transmittance estimation architecture.

The initial transmittance is first calculated using the traditional dark channel algorithm. The sky region is judged to be sky region or not according to the sky region mask. The non-sky region transmittance is unchanged; the sky region uses the tolerance Q to calculate the sky region transmittance correction parameter and make a correction to the transmittance. The transmittance of the whole image is obtained by combining the two regions. The logical architecture is shown in Figure 9.

#### 4.2.3. Image Defogging Module

The input data of the image defogging module include the color image Img, transmittance t(x), and the calculated atmospheric light value A. The defogging module is designed according to Equation (6), and in order to avoid the negative number of the image pixel value subtracted from the atmospheric light value to produce a computational error, the algorithmic circuit design needs to compare the pixel value of the current point to determine the atmospheric light value. When the pixel value is greater than the atmospheric light value, i.e., I≥A, Equation (6) remains unchanged; if I<A, Equation (6) can be transformed into

(13) $$J\left(x\right)=A-\frac{A-I\left(x\right)}{\max\left(t\left(x\right),t_{0}\right)}$$

**Figure 10:**
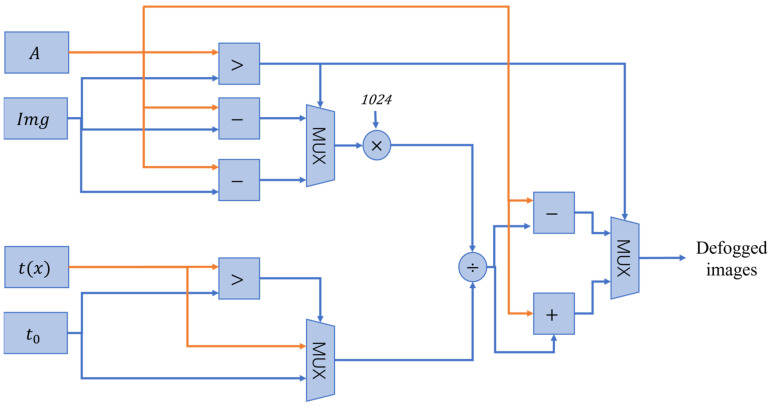
Architecture of defogging module.

In order to reduce the floating-point operation in the operation, it is necessary to shift the value of transmittance, the difference between the atmospheric light value and the original image, to the left operation, and then shift the recovery to the right after the calculation to complete the image defogging. The module architecture is shown in Figure 10.

#### 4.2.4. Color Recovery Module

**Figure 11:**
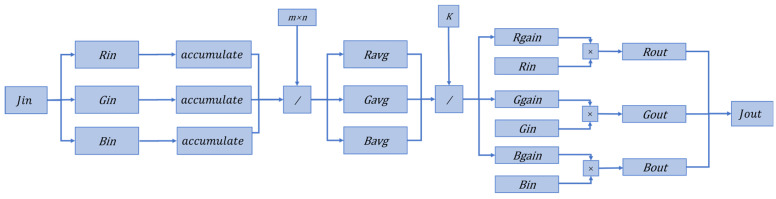
Color recovery module design.

First accumulate the grayscale values of the three channels. Define three accumulators to add the gray values of each pixel in the three channels. Here you need to pay attention to the accumulator bit width—there cannot be overflow. Divide the result by the number of pixels to establish the average gray value of the three channels, $R_{avg}$, $G_{avg}$, $B_{avg}$, and use the threshold K to determine the respective gain of the three channels, $R_{gain}$, $G_{gain}$, $B_{gain}$, and finally multiply each pixel by its respective channel gain to obtain the white balance result. Figure 11 shows the hardware implementation of this module.

FPGA takes a lot of time and arithmetic units to complete the division operation. Xilinx’s FPGA internal DSP can do multiplication quickly. Consider using multiplication operation to implement division, i.e., calculating the mean value of each channel by multiplying by the reciprocal of the number of pixels. Since the reciprocal is a decimal number, the FPGA fixed-point operation is used to fix the decimal places. In the test, the resolution of the image is: 454 × 302 = 137,108. In total, 32 bits of fixed point are taken, one integer and 31 decimals. The inverse of the resolution is 1/137108=0.0000072935204364442629168. Convert the inverse to a hexadecimal. Truncate and select the high 32 bits to obtain 0x00003D2E. Convert this number to a decimal as 0.000007293187081813812255859375. The error generated after the truncation is within the acceptable range. In practical applications, a lookup table is used to implement the inverse calculation module to avoid a waste of resources.

## 5. Hardware Optimization

### 5.1. Image Window Filter Design

**Figure 12:**
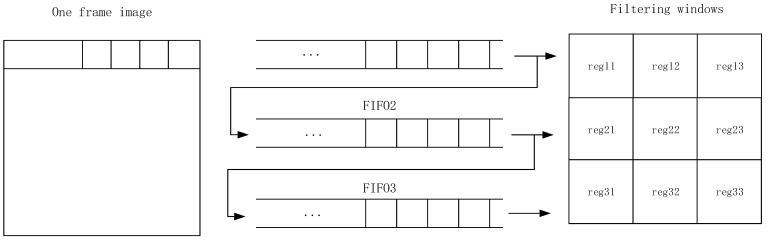
Window filtering principle.

The PL (FPGA) utilizes an image-filtering algorithm by making a sliding filter out of the image data using a filter template, which requires a buffer area to be opened within the FPGA to form an image window, and a single line of data of the image to be cached using the FIFO IP core. Take the 3 × 3 window as an example. The realization principle is shown in Figure 12.

The operations in the algorithm such as finding the gradient of the image, the mean, maximum and minimum values of the pixels in the window are performed using this structure.

### 5.2. Pipeline Computing Architecture Design

**Figure 13:**
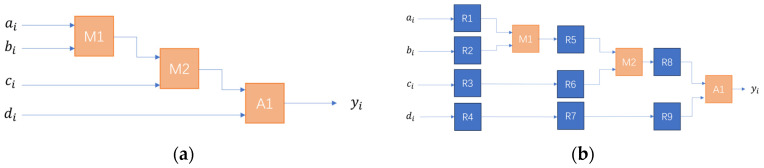
Comparison with normal circuit and pipeline structure: (**a**) original circuit design; (**b**) flow line structure.

The design of the defogging algorithm circuit module is complex, and the combinational logic will cause a large delay and cannot meet the pixel clock. Therefore, the circuit is optimized using a pipelined parallel structure. The core is to utilize registers to temporarily store intermediate data. Time acceleration is traded by increasing the number of registers. The hardware circuit is split into several equalized stages, and by inserting registers to temporarily store intermediate data at each level of operation, the output result will be held for one clock cycle. This approach delays the output of the first data by two clock cycles, but after that the data no longer add delay. Therefore, the advantage is that the intermediate logic operations are cached once per cycle, which improves the overall clock frequency of the system. As shown in Figure 13, (a) is the original circuit design, M is the adder, A is the multiplier and R is the register. The pipeline design is shown in Figure 13b with the inclusion of multiple register levels.

**Figure 14:**
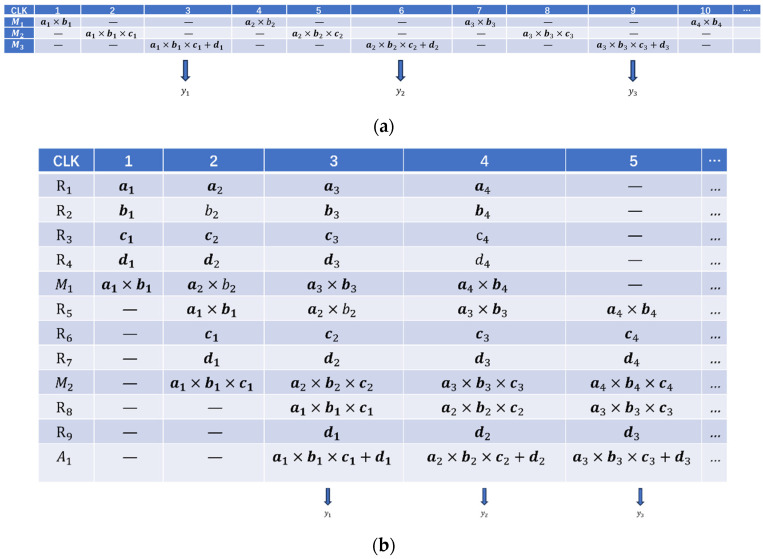
(**a**) Process of original circuit arithmetic; (**b**) process of pipeline structure.

Figure 14 demonstrates the advantage of pipelining, where structure (a) can only output new computation results at the third, sixth, and ninth clock cycles. In contrast, the pipelined structure (b) outputs new computation results at every clock cycle starting from the third cycle.

In this design, the functional design is first completed and then optimized using the pipeline. The combinational logic in the circuit design causes large delays and this part is split. The hardware circuit is split into several equalized stages to increase the overall speed of the system by inserting registers at each level of operation. In addition, due to the complexity of the circuit structure, the HLS tool that comes with the development environment is used to aid in the optimization. The HLS instructions are used to optimize more details and avoid timing problems caused by incorrectly inserted registers.

## 6. Experimental Results and Analysis

### 6.1. Experimental Platforms

**Figure 15:**
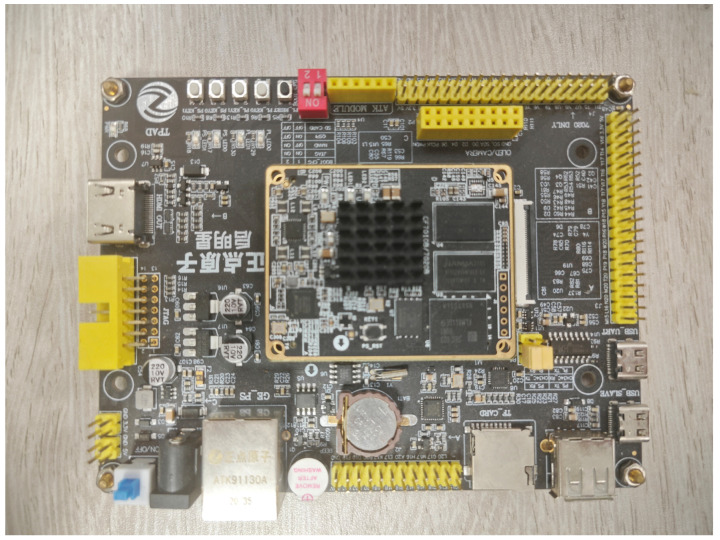
ZYNQ for experiment.

In this article, we use the *VIVADO2018* development environment to realize the design based on the ZYNQ platform. ZYNQ is an all-programmable SoC that consists of PS (Processing System) and PL (Programmable Logic) components. The Zynq SoC integrates the ARM dual-core cortex-A9 processor with the Xilinx 7-series FPGA architecture, giving it not only the power, performance, and compatibility advantages of an ASIC, but also the hardware programmability of an FPGA. The core experimental platform is shown in Figure 15.

### 6.2. Experimental Results and Analysis

#### 6.2.1. Evaluation Indicators

Information entropy, Peak Signal to Noise Ratio (PSNR), Average Gradient (AG), Fog Aware Density Evaluator (FADE), and Structural Similarity Index Measure (SSIM) were chosen as the evaluation indicators for the algorithm’s effect. FADE is an important evaluation indicator for defogging algorithms. The defogging algorithm can be viewed as an algorithm for image restoration. Other indicators are often used in the evaluation of image restoration effects. Therefore, this article chose these indicators for its experiments.

1. Information entropy

The information entropy (IE) represents the amount of information in the image and is calculated as follows:

(14) $$H=\sum_{i=1}^{255}h\left(i\right)logh\left(i\right)$$

H is the information entropy and h(i) is the proportion of grayscale value i, which is used to reflect the average amount of information in the image. A larger value indicates a greater average amount of information in the image. Information entropy is an important evaluation criterion for measuring the effectiveness of image restoration.

2. Peak Signal to Noise Ratio (PSNR) [16]

Suppose there are two images I and *J* of size m×n. Mean squared error (MSE) is defined as follows:

(15) $$\mathrm{MSE}=\frac{1}{\mathrm{mn}}\sum_{i=0}^{m-1}\sum_{j=0}^{n-1}{\left[I(i,j)-J(i,j)\right]}^{2}$$

PSNR can be calculated using MSE:

(16) $$PSNR=20\mathrm{lg}\left(\frac{MAX_{I}}{MSE}\right)$$

MAX is the maximum pixel value, and the smaller the MSE, the larger the PSNR and the lower the level of distortion. However, it is important to note that images with better recovery may have a lower PSNR. Therefore, PSNR needs to be used in combination with other evaluation indicators.

3. Average Gradient

Average Gradient (AG) reflects the image details and texture variations; the larger it is, the richer the texture details. The expression is

(17) $$AG=\frac{1}{mn}\sum_{i=1}^{M}\sum_{j=1}^{N}\sqrt{\frac{{\left(\frac{\partial f}{\partial x}\right)}^{2}+{\left(\frac{\partial f}{\partial y}\right)}^{2}}{2}}$$

∂f/∂x and ∂f/∂y are the horizontal and vertical gradients. The limitation of this parameter is that color distortion can cause the values to be too large.

4. Fog Aware Density Evaluator (FADE)

FADE is an evaluation model proposed by Kwon et al. [17]. It learns the haze image features and accurately evaluates the haze concentration by summarizing the statistical law of measurable deviation between natural images with and without haze. A smaller value represents a better image dehaze effect.

5. Structural Similarity Index Measure (SSIM)

SSIM is used to analyze the degree of information retention of the image before and after processing in three aspects: brightness, contrast, and structure. The larger the value, the more the processed image retains the three aspects of information, and the less distortion there is.

The formula is

(18) $$SSIM\left(x,y\right)=\frac{\left(2\mu_{x}\mu_{y}+c_{1}\right)\left(2\sigma_{xy}+c_{2}\right)}{\left(\mu_{x}^{2}+\mu_{y}^{2}+c_{1}\right)\left(\sigma_{x}^{2}+\sigma_{y}^{2}+c_{2}\right)}$$

x,y are two images; $\mu_{x}$, $\mu_{y}$ are the mean of x,y, respectively; $\sigma_{x}^{2}$, $\sigma_{y}^{2}$ are the variance of x,y, respectively; $\sigma_{xy}$ is the covariance of x and y. $c_{1}$ and $c_{2}$ are constants used to maintain stability.

#### 6.2.2. Qualitative Inorganic Experiment

**Figure 16:**
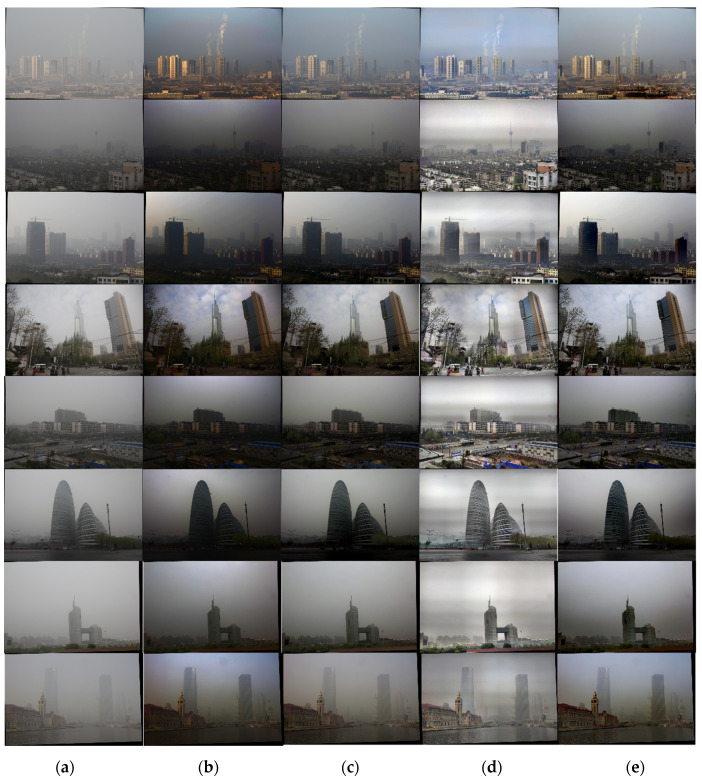
Comparison of image defogging effects: (**a**) original foggy image; (**b**) defogging results of He’s algorithm; (**c**) defogging results of Zhu’s algorithm; (**d**) defogging results of the Retinex algorithm; (**e**) results of the proposed algorithm defogging.

In this article, we use the dataset BeDDE provided in the literature [18]. We use eight sets of foggy images containing sky regions taken in different cities in foggy weather to compare and analyze the effect of He’s algorithm [5], Zhu’s algorithm [19], the Retinex algorithm [11] deployed on FPGA, and this article’s defogging algorithm. He’s algorithm is the original dark channel algorithm. Zhu’s algorithm is a good optimization of the dark channel algorithm. The Retinex algorithm is similar to the proposed algorithm, which is deployed on the FPGA to take full advantage of the speed of the hardware. The experiment results are as shown in Figure 16.

Foggy images containing a large number of sky regions at several locations in China were selected for the experiment. It can be seen that He’s algorithm is effective in removing fog, but the images have the problem of darker brightness and the halo phenomenon in part of the sky area. The Retinex algorithm has a different theoretical starting point from He’s algorithm, which avoids the problem of reduced brightness, but the images have more obvious stripes of light halos and color distortion. The picture quality is poor. Zhu’s algorithm improves on He’s algorithm, which has some improvement in the brightness and the avoidance of halo phenomenon, but relatively weaker fog removal effect can still be seen. Zhu’s algorithm is an improvement on He’s algorithm, which shows some improvement in brightness and avoidance of the halo phenomenon, but a relatively weakened the defogging effect, and obvious fog can still be seen. In this article, the algorithm improves the brightness of the picture after defogging while ensuring the effect of fog removal, attenuates the halo phenomenon and color aberration, and retains more image details at the same time.

#### 6.2.3. Quantitative Experiment

Information entropy, AG, PSNR, FADE, and SSIM are utilized to evaluate the defogging effect, as shown in Table 2.

As can be seen from Figure 16 and Table 2, the FADE of He’s algorithm processing image is improved greatly, but the actual brightness of the image is too dark; the information entropy of Zhu’s algorithm is improved on the basis of He, and the effect of defogging in some scenes is better than that of He’s algorithm, and the indexes of PSNR, FADE, and SSIM are all improved compared with He’s algorithm. The Retinex algorithm performs the best in terms of the average gradient, and at the same time, the information entropy of the scene is better, but this is due to the presence of color distortion and serious halo phenomenon in the image. Compared with the original image, the information entropy of the defogging result of the proposed algorithm is the best, and only some scenes are slightly lower than that of the Retinex algorithm; at the same time, in the case of ensuring that the image is not distorted and retaining more details, the FADE parameter index is the lowest, and the overall defogging effect is better.

### 6.3. Practical Application Experiment

**Figure 17:**
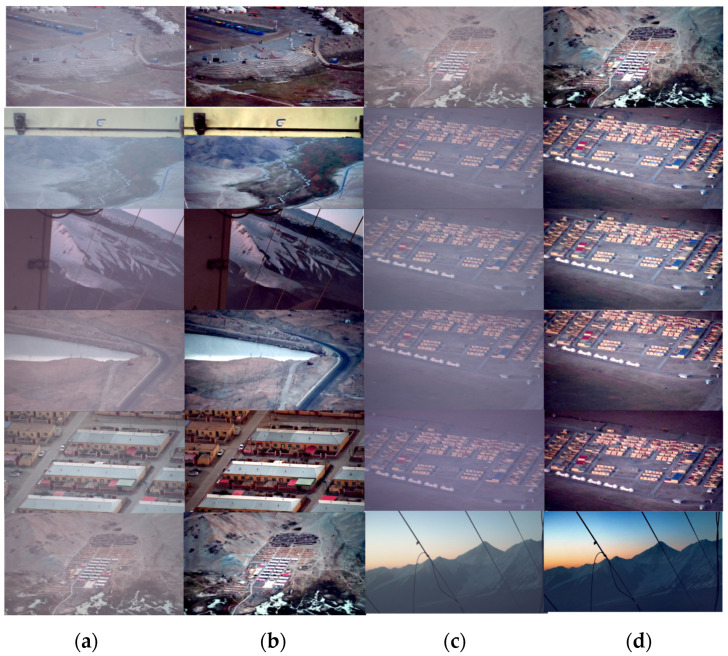
Results of the video defogging experiment: (**a**) original video frame; (**b**) defogging results of (**a**); (**c**) original video frame; (**d**) defogging results of (**c**).

Experiments were conducted using this design in practical applications. The experimental equipment was connected to a visible light camera at a high altitude and the high-altitude video captured by the camera was processed in real time. A computer on the ground acquired both the initial video and the algorithmically processed video. Figure 17 shows the defogging effect on different targets and different frames. In the practical application, it was found that the device defogging effect was significant. During the experiment, the power of the experimental equipment did not exceed 12 W (12 V/1 A). The delay between the returned original video and the defogged video was basically negligible compared to the original video, which meets the requirements of real-time processing and observation.

The comparison of Figure 17 shows that the quality of the defogged image is greatly improved. Comprehensive analysis shows that the real-time defogging system designed in this paper has a good processing effect, and for images containing the sky region in different scenes, the picture recovery effect is good, and through the human eye it can be found that the color, contrast, and clarity of the image have been greatly improved. This system is strong in real time and can be applied to real-time defogging scene applications. The equipment works well in high-altitude scenes at different angles and distances.

## 7. Conclusions

In this article, a real-time defogging system based on sky segmentation is designed by analyzing the shortcomings of traditional dark channel defogging algorithms and the characteristics of parallel advantages of the ZYNQ platform. The grayscale and gradient characteristics of the sky region are first used to obtain the sky and non-sky regions, and then the calculation methods for atmospheric light and transmittance are corrected. The modified parameters are utilized for image defogging. Finally, the algorithm is implemented on the ZYNQ hardware platform. By comparing a variety of defogging algorithms, the experimental results show that the defogging system adapted to the ZYNQ platform proposed in this article has low power consumption, high real-time performance, and a significant defogging effect, which provides a foggy image optimization scheme with a wider range of applications.

## Figures and Tables

**Table 1:** Sub-module description of the defogging module.

Submodule Name	Functional Description	Input and Output Description
Sky Split Module	Splitting of the sky area	Input image, output segmented binary data
Atmospheric Light Estimation Module	Calculate atmospheric light	Input RGB image and segmented binary data, output atmospheric light estimate
Transmittance Estimation Module	Calculate transmittance	Input RGB image, atmospheric light value, segmented binary data, output transmittance
Image Defogging Module	Image Defogging	Input RGB image, transmittance, atmospheric light value, output defogging image
Color Recovery Module	White balance and color recovery	Input defogged image, output color restored image

**Table 2:** Evaluation of defogging image parameters.

Location	Algorithm	IE	AG	PSNR	FADE	SSIM
Lanzhou	original	5.8723	1.579	/	5.3041	/
	He	6.8910	3.7979	14.2081	1.7291	0.6850
	Zhu	6.7022	2.8417	19.2824	0.6680	0.7031
	Retinex	7.0603	3.5633	20.9224	1.8821	0.4483
	proposed	7.3431	3.1376	16.5864	0.5786	0.6951
Chengdu	original	6.7493	2.0676	/	2.4586	/
	He	6.7301	2.5304	15.0803	1.1993	0.6962
	Zhu	6.9432	2.628	20.3080	1.3677	0.8990
	Retinex	7.7157	7.295	12.1497	0.8875	0.6555
	proposed	7.3467	3.5013	18.3036	0.7977	0.7682
Nanchang	original	7.2685	1.995	/	3.6027	/
	He	7.1825	2.4205	12.2666	1.4509	0.6741
	Zhu	7.5062	2.6928	14.4561	1.1892	0.7542
	Retinex	7.7210	4.0853	19.0270	1.6263	0.8277
	proposed	7.7710	3.1436	15.7339	1.2038	0.7521
Nanjing	original	7.3903	5.9233	/	1.2922	/
	He	7.3074	5.2014	13.6277	0.7078	0.6498
	Zhu	7.4411	5.9445	16.1896	0.6680	0.7823
	Retinex	7.7474	10.9883	17.9105	0.7023	0.7894
	proposed	7.6409	9.5108	18.9955	0.5786	0.7255
Hangzhou	original	7.1206	2.3443	/	2.2199	/
	He	6.7988	2.4548	14.3508	1.0598	0.6550
	Zhu	7.0579	2.6500	17.2248	0.9674	0.7702
	Retinex	7.8312	6.1488	15.3961	0.9038	0.7451
	proposed	7.5173	3.4645	17.9642	0.7954	0.7597
Beijing	original	7.1474	2.0687	/	2.9377	/
	He	6.9726	2.7313	13.8229	1.2686	0.6738
	Zhu	7.2241	2.5025	16.8757	0.9622	0.7371
	Retinex	7.6815	4.1496	18.4141	1.4905	0.8408
	proposed	7.5604	3.0022	17.4263	1.1061	0.7962
Changsha	original	6.7011	1.2792	/	4.9663	/
	He	7.0550	2.5069	13.2670	1.8195	0.7730
	Zhu	7.2705	2.0447	17.3320	2.4672	0.9073
	Retinex	7.3400	2.5568	20.0884	2.4456	0.8342
	proposed	7.5243	1.7719	15.4866	1.9376	0.7731
Tianjin	original	6.5346	1.5423	/	3.6766	/
	He	7.0202	3.0832	15.3363	1.2330	0.6743
	Zhu	6.9932	2.3151	19.8832	1.9124	0.8699
	Retinex	7.0140	2.8915	24.4179	1.9765	0.8564
	proposed	7.3762	2.3734	19.7190	1.3998	0.7178

## Data Availability

The BeDDE data and exBeDDE data were downloaded from the Github and the internet URL is https://github.com/xiaofeng94/BeDDE-for-defogging (accessed on 12 November 2023). The data that support the findings of this study are available from the corresponding author upon reasonable request.
